# Dr. Leonard R. Koecker

**Published:** 1896-06

**Authors:** 


### DR. LEONARD R. KOECKER.

Dr. Leonard R. Koecker died May 6, 1896, at his residence, 1302 Walnut Street, Philadelphia, after a short illness. Very few, it is presumed, in the dental profession, even in Philadelphia, were familiar with Dr. Koecker except by name. He has not taken any active part in dentistry outside of practice for many years.

Dr. Koecker was born in Philadelphia, July 16, 1822. His father was the celebrated Dr. Koecker, who, it is believed, wrote the first book on dentistry published in the United States, and so frequently quoted in later works. The son, after graduating in medicine at the Jefferson Medical College, in 1842, chose the practice of dentistry as a profession, and continued in this as his life work, retiring from active practice some ten years since, thereafter devoting himself principally to literary pursuits.

For years Dr. Koecker has been known as a collector of rare and valuable prints, and a choice collection of his is the nucleus of the famous Claghorn collection, now owned by Mr. Walters, of Baltimore.

It is said of him that his reputation as a wood-, paper-, and metal-worker long ago extended beyond this country, and that some of the finest gems in bookbinding and illustrating ever seen were exhibited by him at the Centennial Exhibition in Philadelphia in 1876. It is also said of him that as a metal-worker he was frequently called upon by the United States government to decide questions of great delicacy involved in the construction of intricate and sensitive pieces of machinery.

A widow and two children survive him.

